# A Review of 177Lutetium-PSMA and 225Actinium-PSMA as Emerging Theranostic Agents in Prostate Cancer

**DOI:** 10.7759/cureus.29369

**Published:** 2022-09-20

**Authors:** Mohammad R Alam, Shashi B Singh, Shreeya Thapaliya, Shreeya Shrestha, Sulav Deo, Kishor Khanal

**Affiliations:** 1 Department of Internal Medicine, Argakhachi Hospital Pvt. Ltd, Sandhikharka, NPL; 2 Department of Radiology, KIST Medical College and Teaching Hospital, Kathmandu, NPL; 3 Department of Internal Medicine, Kathmandu Medical College Teaching Hospital, Kathmandu, NPL; 4 Department of Internal Medicine, Manipal College of Medical Sciences, Pokhara, NPL; 5 Department of Internal Medicine, Suraksha Hospital, Biratnagar, NPL; 6 Department of Cardiology, Memorial Healthcare System, Pembroke Pines, USA

**Keywords:** [18f]fdg, 177lu-psma, 225ac-psma, pet/ct, psma, prostate-specific membrane antigen, prostate cancer

## Abstract

The development of prostate-specific membrane antigen (PSMA) ligands labeled with radionuclides is a ground-breaking achievement in the management of prostate cancer. With the increasing use of ^68^Gallium-PSMA and ^18^F-DCFPyL (Pylarify) and their approval by the Food and Drug Administration (FDA), other PSMA agents and their unique characteristics are also being studied. Two other PSMA agents, namely ^177^Lutetium-PSMA (^177^Lu-PSMA) and ^225^Actinium-PSMA (^225^Ac-PSMA), are currently drawing the researcher’s attention mainly due to their theranostic importance. Studies focusing on the essential characteristics of these two emerging radiotracers are relatively lacking. Hence, this review article, beginning with a brief introduction, intends to provide insights on the mechanism, efficacy, adverse effects, usefulness, including theranostic implications, and limitations of these two emerging PSMA agents. The ^177^Lu-PSMA is commercially accessible, is well tolerated, and has been found to lower prostate-specific antigen (PSA) levels while improving patients’ quality of life. It also reduces pain and the requirement for analgesics and is safe for advanced diseases. However, despite its potential advantages, around one-third of patients do not respond satisfactorily to this costly treatment; it is still challenging to personalize this therapy and predict its outcome. Similarly,^ 225^Ac is compatible with antibody-based targeting vectors, releasing four extremely hazardous high-energy emissions with a longer half-life of 10 days. It has made ^225^Ac-PSMA therapy useful for tumors resistant to standard treatments, with a better response than ^177^Lu-PSMA. Dosimetry studies show a good biochemical response without toxicity in patients with advanced metastatic castration-resistant prostate cancer (mCRPC). However, it can potentially cause significant damage to healthy tissues if not retained at the tumor site. Encapsulating radionuclides in a nano-carrier, hastening the absorption by tumor cells, and local delivery might all help reduce the harmful consequences. Both have advantages and disadvantages. The choice of PSMA agents may rely on desired qualities, cost, and convenience, among other factors. Further research is warranted in order to better understand their ideal use in clinical settings.

## Introduction and background

Prostate cancer is the most prevalent cancer among American men, after skin cancer. The American Cancer Society (ACS) estimates that approximately one in eight men will be diagnosed with prostate cancer in their lifetime. It is more prevalent in older men and non-Hispanic black men, with an average age of 66 years at diagnosis. Moreover, it is the second leading cause of cancer-related mortality among American men after lung cancer. Approximately one in 41 men will eventually die from prostate cancer, according to an estimate by ACS [1]. Prostate cancer can be deadly, but most men diagnosed with it do not succumb to it. More than 3.1 million men diagnosed with prostate cancer in the United States are still alive today [1]. Prostate cancer is generally indolent, with numerous treatment options such as androgen deprivation therapy, radical surgical resection, radiotherapy, chemotherapy, and immunotherapy. However, its prognosis becomes poor when it aggressively metastasizes despite the initial treatment, eventually progressing into metastatic castration-resistant prostate cancer (mCRPC) [2]. Here comes the emerging role of prostate-specific membrane antigen (PSMA)-targeted radioligand therapy (PRLT) as a promising treatment modality for managing mCRPC. Several small-molecule PSMA ligands that can be conjugated to radioisotopes, such as ^18^F, ^68^Ga, ^177^Lu, and ^225^Ac, among others, have been developed [3]. FDA approved 68Gallium PSMA (^68^Ga-PSMA) in 2020, and [^18^F]DCFPyL, a Fluorine-18 labeled PSMA ligand, popularly known as Pylarify in 2021 as the first and second PSMA-based PET tracer for management of patients with prostate cancer who had a biochemical recurrence. Recently, PSMA-based radiotracers such as 177Lutetium-PSMA (^177^Lu-PSMA) and ^225^Actinium-PSMA (^225^Ac-PSMA) have become available with unique diagnostic and therapeutic benefits. It involves using PSMA molecules radiolabeled with the beta (β) and gamma (γ) emitters) such as ^177^Lu and the α-emitter such as ^225^Ac. The ^177^Lu emits a cytotoxic β-particle useful for targeted therapies and γ-particles whose emissions can be quantified to assist with the diagnostic evaluation [4]. Similarly, targeted alpha-particle therapy (TAT) uses alpha-particles released by ^225^Ac and can potentially treat metastases in soft tissues [5,6]. In light of the rapidly evolving adoption of [^18^F]DCFPyL and ^68^Ga-PSMA globally, there is a relative scarcity of literature to highlight the advantages and limitations of ^177^Lu-PSMA and ^225^Ac-PSMA, which are currently being studied extensively. This review article aims to shed light on the pros and cons of these two emerging PSMA agents, ^177^Lu-PSMA and ^225^Ac-PSMA, which are gradually gaining popularity, mainly due to their theranostic importance.

## Review

^177^Lu-PSMA

^177^Lu-PSMA-617 radioligand therapy ([RLT] which is regarded as the mainstay ^177^Lu-labeled PSMA agent in this review) has demonstrated its ability to target prostate cancer cells while sparing most normal tissues in patients that have been identified using imaging to confirm radionuclide binding with the prostate cancer cells [7]. Anderson et al. reported the first clinical use of ^177^Lu in 1960 when three patients with myelomatosis were treated with intravenous injections of ^177^Lu as lutetium chloride and citrate [8]. Keeling and Vaughan published a study on ^177^Lu hydroxyapatite (HA) particles to investigate the mechanism of uptake of bone minerals using in vitro techniques in 1988, which was the first publication on ^177^Lu [9]. However, the potential application of ^177^Lu as a therapeutic radionuclide was established with the introduction of ^177^Lu-DOTATATE, a radiopharmaceutical that targets neuroendocrine tumors [10,11]. The significant rise in interest in ^177^Lu as a therapeutic radionuclide can be due to its favorable nuclear properties and adaptable chemistry, which results in stable compounds with good in vivo properties. However, the ease with which ^177^Lu may be produced in high activity levels with high specific activity in many current nuclear reactors worldwide is the single most crucial reason contributing to its rising interest and use in nuclear medicine [12]

Mechanism of ^177^Lu-PSMA

**Figure 1:**
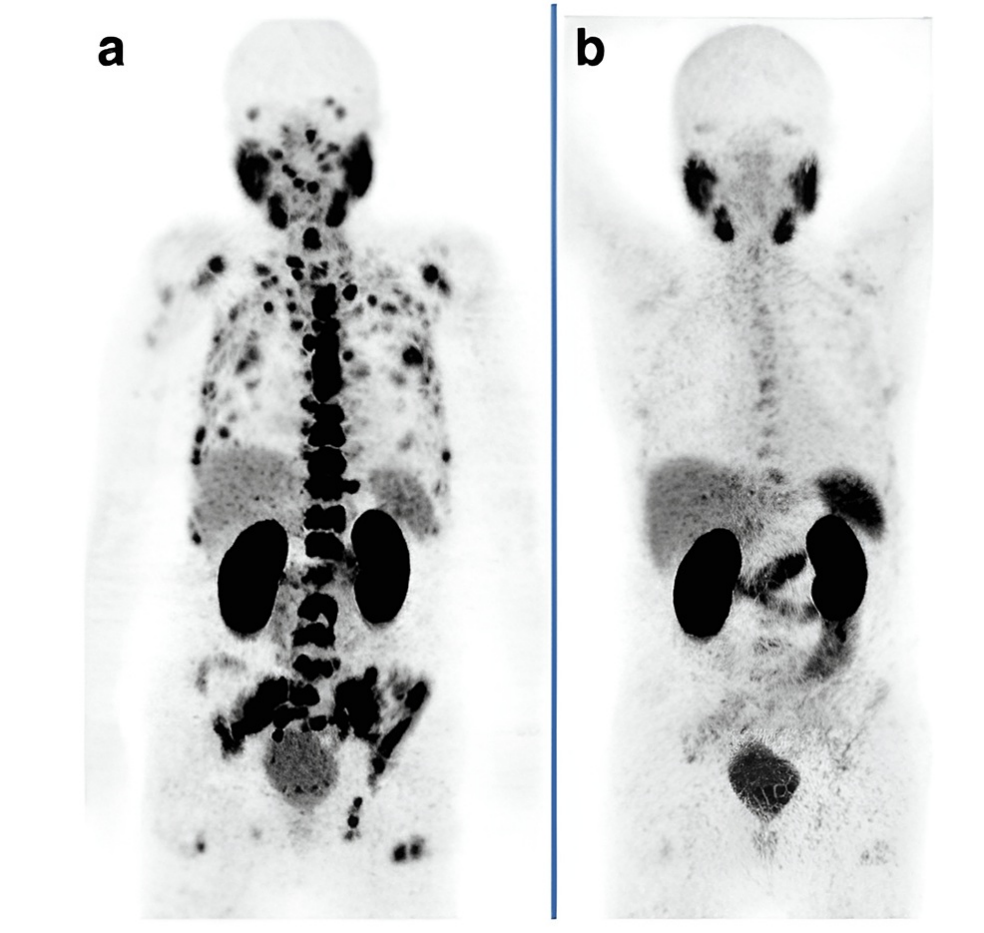
Efficacy of 177Lutetium prostate-specific membrane antigen. An 83-year-old patient with castration-resistant prostate cancer (Gleason score: 9) and an increasing prostate-specific antigen (PSA) level. He had a history of prostatectomy and radiation therapy on the prostate bed. The ^68^Ga-PSMA PET scan showed diffuse bone and bone marrow involvement (a). Before the first cycle of ^177^Lu-PSMA therapy, PSA and alkaline phosphatase (ALP) levels were 261 ng/mL and 659 U/L, respectively. The patient received two cycles of ^177^Lu-PSMA, and the PSA level decreased continuously during cycles from 261 to 9.0 ng/mL (eight weeks after the second cycle). The ALP also reduced from 659 to 81 U/L (eight weeks after the second cycle). PSMA-PET (b) eight weeks after the second cycle showed a significant response with significant regression of PSMA. This research was originally published in Radiation Oncology. Awang ZH, Essler M, Ahmadzadehfar H. Radioligand therapy of metastatic castration-resistant prostate cancer: current approaches. Radiation Oncology. 2018;13(1):1-9 [13]

The beta-particle radiation delivered by ^177^Lu-PSMA-617 is preferential to PSMA-positive cells and the tissues surrounding them [4,12]. The internalization of the radioligand makes it possible for radioactivity to accumulate in the tumor tissue, allowing for inside-out irradiation. ^177^Lu-PSMA-617 has a half-life of six to eight days [8]. Figure 1 demonstrates its efficacy in prostate cancer.

^177^Lu-PSMA RLT appears safe, even in patients with advanced disease [13]. Because most prostate cancer patients had repeated relapses before receiving PSMA-RLT therapy, the results are pretty encouraging [11,14,15]. An average of 7.5 to 15 months is recorded for overall survival (OS) and 4.5 to 13.7 months for progression-free survival (PFS) [16]. Important findings of studies assessing OS and objective remission (complete remission and partial remission) are presented in Table 1. However, it is worth noting that the response rate is fluctuating [16]. Nearly one-third of patients do not respond satisfactorily to this expensive treatment. At the later stages of the disease, the therapy options available to individuals who have experienced a relapse following PSMA-RLT are much restricted. This demands further individualization of therapy and better prediction of clinical outcomes [11].

**Table 1:** Overview of overall survival and objective remission of 177Lutetium prostate-specific membrane antigen. *Complete remission and partial remission NR, not reported

Author	Total number of patients	Frequency of best PSA decline of ≥50% (%)	Frequency of objective remission* (%)	Median overall survival (months)
Kratochwil et al. [17]	30	43%	NR	NR
Kulkarni et al. [15]	119	58%	29%	70% at 15 months
Ahmadzadehfar et al. [18]	22	60%	NR	14
Brauer et al. [19]	59	53%	NR	8
Fendler et al. [20]	15	47%	27%	NR
Rahbar et al. [21]	145	49%	NR	NR
Rahbar et al. [22]	104	33%	NR	14
Peters and Stahel [23]	10	50%	30%	NR
Yadav et al. [24]	31	71%	82%	16
Khreish et al. [25]	252	48%	NR	13.4
Sartor et al. [26]	551	46%	NR	15.3
Hofman et al. [27]	98	66%	NR	NR

Adverse effects of ^177^Lu-PSMA 

Anemia, neutropenia, and thrombocytopenia were some treatment-induced hematologic toxicities reported in a prospective study by Hofman et al. [27]. Extensive bone marrow metastases, previous chemotherapy, and older patients (likely associated with reduced renal function) are all factors that might amplify hematopoietic damage. Patients with renal impairment should get a modified activity dosage to protect the red marrow [28]. Transient xerostomia is a treatment-related side effect that may have a negative impact on quality of life. Nephrotoxicity can occur. However, severe cases of renal failure are rare [14,24]. According to the findings of Barber et al., grade I-II renal toxicity (as determined by estimated glomerular filtration rate) was seen in 42/167 individuals; 26 of these patients had reduced baseline renal function. Not a single patient reported having grade III-IV renal toxicity [29]. In their series, many additional investigations found no evidence of treatment-induced nephrotoxicity [30-32]. Old age, systemic hypertension, and pre-existing renal impairment are risk factors for ^177^Lu-PSMA renal toxicity [33]. A case of tumor lysis syndrome due to ^177^Lu-PSMA treatment has been reported [34]. Table 2 illustrates the percentage of occurrence of these common side effects in various studies.

**Table 2:** Overview of adverse effects of 177Lutetium prostate-specific membrane antigen in some published studies. NR, not reported

Study	Total number of patients	Hematologic toxicity			Nephrotoxicity	Salivary gland toxicity
		Anemia	Neutropenia	Thrombocytopenia		
Ahmadzaehfar et al. [35]	24	38%	21%	17%	12.5%	9%
Baum et al. [36]	56	5%	16%	0%	0%	4%
Heck et al. [37]	22	32%	5%	25%	NR	37%
Kratochwil et al. [17]	30	10%	27%	7%	0%	7%
Kulkarni et al. [17]	119	4%	NR	NR	NR	NR
Rahbar et al. [38]	74	36%	16%	23%	5.4%	9%
Rahbar et al. [39]	28	20%	11%	23%	4.5%	0%
Brauer et al. [19]	59	85%	38%	47%	85%	85%
Yadav et al. [24]	31	7%	3%	0%	0%	0%
Hofman et al. [40]	30	26%	30%	30%	NR	87%

Limitations of ^177^Lu-PSMA 

According to the findings of a recent meta-analysis, around 37% of patients show biochemical progression and are resistant to the treatment provided by ^177^Lu-PSMA-617 [41]. Alternative treatment options are required for patients who do not respond to ^177^Lu-PSMA and are unfit or exhausted from receiving the approved therapies. Additionally, many patients who respond to ^177^Lu-PSMA will ultimately progress. Patients whose prostate cancer has spread throughout the bone marrow and has induced bone marrow failure may not be good candidates for ^177^Lu-PSMA due to the extended route length of ^177^Lu, which may cross 20 to 60 cells resulting in bone marrow failure [42]. This necessitates the understanding of major properties of alternative PSMA agents such as ^225^Ac-PSMA.

^225^Ac-PSMA

Although alpha emitters can be more efficacious than beta emitters, only a limited number of alpha-emitting radionuclides are commercially accessible and possess the necessary properties for use in medical settings. In the last decade, the radioactive metals ^225^Actinium (t1/2 = 10 days) and 227Thorium (t1/2 = 19 days) have emerged as potentially useful alpha emitters that might be used in the development of future targeted radiotherapeutics [43].

TAT is a new approach aiming to take advantage of alpha-particles therapeutic potential for metastases in soft tissues [5,6]. To selectively administer cytotoxic alpha radiation to cancer cells, radionuclides that generate alpha radiation are coupled to tumor-targeting vectors utilizing bifunctional chelators [44]. ^225^Ac, one of the radionuclides suitable for such an application, has a long half-life of 10 days, is compatible with antibody-based targeting vectors, and emits four high-energy emissions that are very harmful to cells. This makes it a better option for use in TAT. ^225^Ac is easily complexable with the DOTA chelator under temperatures of 80-90°C, which can be achieved by microwave or other methods [45]. But initial research suggested that the chelator macropa (mcp) would be even more appropriate [46]. Additionally, ^213^Bismuth is produced when ^225^Ac decays, and this latter compound also emits 440 keV rays, which may be used to image the therapeutic biodistribution. It should be noted that it is unclear if the detected radioactive decay reflects liberated daughter radioisotopes or intact radiopharmaceuticals [47,48]. The relatively long half-life of ^225^Ac enables centralized manufacturing and shipping of the irradiated targets to other users allowing its widespread use.

Excellent response to ^225^Ac-PSMA was first reported by Kratochwil et al. in two patients who had previously failed ^177^Lu-PSMA treatment [32]. Patients with advanced mCRPC have shown a satisfactory biochemical response and low toxicities with ^225^Ac-PSMA-617 TAT, according to dosimetry studies [49].

Mechanism of ^225^Ac-PSMA

**Figure 2:**
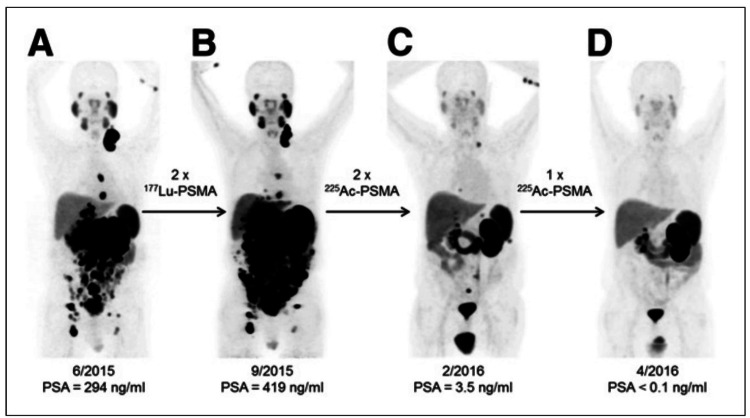
Efficacy of 225Actinium prostate-specific membrane antigen. ^68^Ga-PSMA-11 PET/CT of a patient. Compared to initial tumor spread (A), restaging after two cycles of β-emitting ^177^Lu-PSMA-617 presented progression (B). In contrast, restaging after the second (C) and third (D) cycles of α-emitting ^225^Ac-PSMA-617 gave an impressive response. This research was originally published in Journal of Nuclear Medicine. Kratochwil C, Bruchertseifer F, Giesel FL, Weis M, Verburg FA, Mottaghy F, Kopka K, Apostolidis C, Haberkorn U, Morgenstern A. 225Ac-PSMA-617 for PSMA-targeted α-radiation therapy of metastatic castration-resistant prostate cancer. J Nucl Med. 2016;57(12):1941-4. © by the Society of Nuclear Medicine and Molecular Imaging, Inc. [51]

Alpha emitters such as ^225^Ac-PSMA can treat cancer more effectively than beta emitters such as ^177^Lu-PSMA because of the narrow range of alpha radiation in human tissue (less than 0.1 mm), which corresponds to just a few cell diameters. This, in turn, enables the selective destruction of cancer cells being targeted while preserving the healthy tissues around them. In addition, the high energy of alpha particles, which may be many MeV, along with the strong linear energy transfer that comes with it, causes a significant increase in the number of cells killed. As a consequence, alpha radiation can kill cells that, under normal circumstances, would be resistant to treatment with beta or gamma irradiation or chemotherapeutic drugs, hence making alpha radiation a viable treatment option for tumors that are resistant to conventional therapies [50] (Figure 2).

Efficacy of ^225^Ac-PSMA

The Prostate Cancer Clinical Trials Working Group 3 recommends using a PSA drop of more than 50% as a standard for measuring therapeutic success in patients with mCRPC [52]. Recent meta-analyses by Lee et al. revealed that approximately 61%percent of patients showed more than a 50% PSA decline after receiving ^225^Ac-PSMA RLT and that 84% of patients showed any PSA decline after receiving ^225^Ac-PSMA RLT [53]. It has a greater response than what was shown in a prior meta-analysis for ^177^Lu-PSMA RLT: 46% by Yadav et al. [41] and 57 % by Hofman et al. in a previous phase two clinical trial of ^177^Lu-PSMA-617 [54]. After receiving ^177^Lu-PSMA RLT, the median PFS and OS were found to be 11 months and 14 months, respectively, in a study by Yadav et al. [41]. This is a longer period than what Lee et al. found; the median PFS and OS were 8 months and 12 months, respectively, following ^225^Ac-PSMA RLT [53]. PFS, OS, and other characteristics of some important studies related to ^225^Ac-PSMA included in this review article have been listed in Table 3.

**Table 3:** Overview of overall survival, progression-free survival, and complete response in the recently published literature on 225Actinium prostate-specific membrane antigen targeted alpha-particle treatment.

Author	Total number of patients	Frequency of best PSA decline of ≥50% (%)	Frequency of objective remission (%)	Median overall survival (months)
Kratochwil et al. [55]	14	63%	NR	NR
Kratochwil et al. [56]	38	82%	13%	>12
Sathekge et al. [57]	17	70%	NR	NR
Sathekge et al. [58]	73	65%	NR	18
Khreish et al. [59]	20	49%	21%	12
Yadav et al. [60]	28	39%	9%	17

Adverse effects of ^225^Ac-PSMA

The majority of patients who were treated with ^225^Ac-PSMA reported that xerostomia was the most prevalent adverse effect [56-58]. It was so severe that 10% of participants in previous research gave up on treatment. The treatment dose of ^225^Ac-PSMA was gradually adjusted in subsequent studies based on the patient's ability to tolerate the severity of dry mouth without compromising antitumor activity [57,58,61]. This has resulted in a less severe case of xerostomia being recorded in more recent studies, and no patient has had to stop treatment as a direct result of xerostomia. Sialendoscopy with dilation, isotonic saline irrigation, and steroid injection improved salivary gland function in individuals with intolerable xerostomia [62].

In a study conducted by Feuerecker et al., patients exhibited anemia of grade III/IV, thrombocytopenia, and leukopenia at varying rates: 35%, 19%, and 27%, respectively [63]. Feuerecker et al. [63] documented grade I/II impairment of kidney function in 19% of patients but without clinical relevance, similar to what Sathekge et al. described [58]. The most significant disadvantage associated with ^225^Ac is its high price. In addition, the recoiled daughters of ^225^Ac have the potential to cause significant damage to healthy tissues if they are not retained at the tumor site. Harmful effects produced by the daughters that create alpha particles could be minimized by encapsulation in a nano-carrier, rapid absorption of the radionuclides by tumor cells, and local delivery of the radionuclides to the tumor location [64].

## Conclusions

The discovery of various PSMA ligands labeled with radionuclides is a novel diagnostic and therapeutic option in the management of prostate cancer. Their uses are growing rapidly globally, owing to their diagnostic superiority over other conventional imaging modalities and some PSMA agents having an additional advantage of theranostic value. ^177^Lu-PSMA emits a cytotoxic β-particle useful for targeted therapies and γ-particles whose emissions can be quantified to assist with diagnostic evaluation and dosimetry studies. The ^177^Lu-PRLT (PRLT) has demonstrated its promising results in mCRPC with reductions in PSA level, relief from pain, and reduced need for analgesics. Moreover, it has been found to be superior to other third-line systemic treatments. However, patients with prostate cancer that have metastases to the bone marrow may not be suitable candidates for ^177^Lu-PSMA as ^177^Lu can pass through 20 to 60 cells causing bone marrow failure. ^225^Ac-PSMA, an α-emitter, is another promising agent owing to its ability to kill both single cancer cells and clusters of cancer cells with only minor collateral damage to healthy, non-targeted cells, as shown in both preclinical and clinical studies. There is growing evidence that ^225^Ac-PSMA TAT is superior to ^177^Lu-PSMA RLT in terms of tumor control. However, there is also evidence that co-radiation to the salivary glands is more common with the former. 

Clearly, both of these agents have their own pros and cons. Hence, the choice of PSMA agents might depend on the desired properties of the agent, the cost of treatment, and the convenience of adopting that agent, among others. It is still unknown whether PSMA RLT can benefit patients with nonexistent or low PSMA expression, thereby limiting a suitable and effective therapy plan in this setting. Conducting high-quality, multicenter, prospective, randomized controlled studies to assess the efficacy, safety, and survival benefits of these PSMA-targeted radioligands with one another, as well as with traditional therapies, might help us better understand their ideal clinical utility.
